# Epstein-Barr Virus Interferes with the Amplification of IFNα Secretion by Activating Suppressor of Cytokine Signaling 3 in Primary Human Monocytes

**DOI:** 10.1371/journal.pone.0011908

**Published:** 2010-07-30

**Authors:** François Michaud, François Coulombe, Eric Gaudreault, Carine Paquet-Bouchard, Marek Rola-Pleszczynski, Jean Gosselin

**Affiliations:** 1 Innate Immunology Laboratory, CHUQ Research Center (CHUL) and Department of Molecular Medicine, Faculty of Medicine, Université Laval, Quebec, Quebec, Canada; 2 Immunology Division, Department of Pediatrics, Faculty of Medicine, Université de Sherbrooke, Sherbrooke, Quebec, Canada; Washington University, United States of America

## Abstract

**Background:**

Epstein-Barr virus is recognized to cause lymphoproliferative disorders and is also associated with cancer. Evidence suggests that monocytes are likely to be involved in EBV pathogenesis, especially due to a number of cellular functions altered in EBV-infected monocytes, a process that may affect efficient host defense. Because type I interferons (IFNs) are crucial mediators of host defense against viruses, we investigated the effect of EBV infection on the IFNα pathway in primary human monocytes.

**Methodology/Principal Findings:**

Infection of monocytes with EBV induced IFNα secretion but inhibited the positive feedback loop for the amplification of IFNα. We showed that EBV infection induced the expression of suppressor of cytokine signaling 3 (SOCS3) and, to a lesser extent, SOCS1, two proteins known to interfere with the amplification of IFNα secretion mediated by the JAK/STAT signal transduction pathway. EBV infection correlated with a blockage in the activation of JAK/STAT pathway members and affected the level of phosphorylated IFN regulatory factor 7 (IRF7). Depletion of SOCS3, but not SOCS1, by small interfering RNA (siRNA) abrogated the inhibitory effect of EBV on JAK/STAT pathway activation and significantly restored IFNα secretion. Finally, transfection of monocytes with the viral protein Zta caused the upregulation of SOCS3, an event that could not be recapitulated with mutated Zta.

**Conclusions/Significance:**

We propose that EBV protein Zta activates SOCS3 protein as an immune escape mechanism that both suppresses optimal IFNα secretion by human monocytes and favors a state of type I IFN irresponsiveness in these cells. This immunomodulatory effect is important to better understand the aspects of the immune response to EBV.

## Introduction

Epstein-Barr virus (EBV), a human gamma-herpes virus, persists latently in over 90% of the adult population and is the cause of infectious mononucleosis in a small proportion of carriers. Viral reactivation is responsible for certain rare types of lymphoproliferative disorders and cancers [1]. Although its main target cells are B lymphocytes, EBV can spread to other cell types [2], [3]. Particularly, efficient and sustained replication of EBV particles in primary human monocytes has been confirmed and shown to alter a number of cellular defense mechanisms. For example, EBV can negatively regulate monocyte secretion of TNF-α [4] and MIP-1α [5]. In addition, EBV infection reduces monocyte secretion of the antiviral lipid mediator prostaglandin E_2_ (PGE_2_) by targeting the enzyme cyclooxygenase-2 (COX-2) essential for prostaglandin synthesis [6]. We also reported that EBV infection impairs protein kinase C (PKC) function causing a reduction in monocyte phagocytic activity [6], [7]. Because monocytes were shown to contribute to the spread of EBV infection [8], these data suggest that infection of these cells may have important implications in EBV pathogenesis.

Type I interferons (IFNs) critically contribute to host defense against viral invaders by inducing innate responses and subsequent adaptive immunity. Secreted IFNs function in an autocrine and paracrine fashion to potentiate cellular antiviral mechanisms and limit the replication and spread of the virus [9]. Upon viral sensing by host cells, two members of the interferon regulatory factor (IRF) family, IRF3 and IRF7, mainly activate IFN gene transcription and initiate the first wave of IFN secretion [10]. Subsequent binding of IFNs to their cognate receptor leads to the activation of the JAK/STAT pathway. JAK1 and Tyk2 kinases are constitutively associated with the IFN receptor subunits and upon activation, they phosphorylate each other at critical tyrosine residues within the intracellular domain of the receptor. STAT1 and STAT2 factors are then recruited via the phosphorylated tyrosines, bind the activated receptor and are in turn phosphorylated by JAK1 and Tyk2 [11]. Signaling downstream of the IFN receptors through the JAK/STAT pathway creates a positive feedback loop that prolongs activation of IFN-stimulated genes, mediates a second wave of IFN secretion and leads to the production of antiviral proteins such as 2′-5′-oligoadenylate synthetase and dsRNA-dependent protein kinase R (PKR) [12], [13].

In order to avoid excessive host tissue injury whilst protecting effectively against infectious agents, the immune system features regulatory mechanisms to control the production and response to cytokines. The SOCS family of proteins comprises eight members (SOCS1-7 and CIS) critically involved in this process [14]. SOCS1 and SOCS3 are the best-characterized family members and have both been described to interfere with the response to IFNα [14], [15]. The kinase inhibitory region (KIR) shared by SOCS1 and SOCS3 is sufficient to inhibit JAK tyrosine kinase activity [15]. In addition, SOCS1 has been proposed to target itself and JAK proteins to the microtubule organizing complex (MTOC)-associated 20S proteasome for degradation [16]. Importantly, recent studies have shown that several viruses such as hepatitis C virus (HCV) [17], herpes simplex 1 virus (HSV-1) [18], [19], enterovirus [20] and respiratory syncytial virus (RSV) [21] are capable of inducing expression of SOCS proteins and interfere with the IFN signaling pathway.

In the present study, we hypothesized that impairment in IFNα secretion by primary human monocytes infected with EBV involved the activation of SOCS proteins. We tested this hypothesis by examining SOCS1 and SOCS3 expression in parallel with several aspects of the IFNα pathway in infected cells. We showed that depletion of SOCS3 reduced the EBV-mediated suppression of the IFNα pathway and that the EBV protein Zta (also known as ZEBRA) was implicated in activating SOCS3 expression. Interference with the amplification of IFNα secretion caused by EBV infection may constitute an essential strategy that evolved to evade the antiviral response.

## Results

### EBV interferes with IFNα secretion in human monocytes

**Figure 1:**
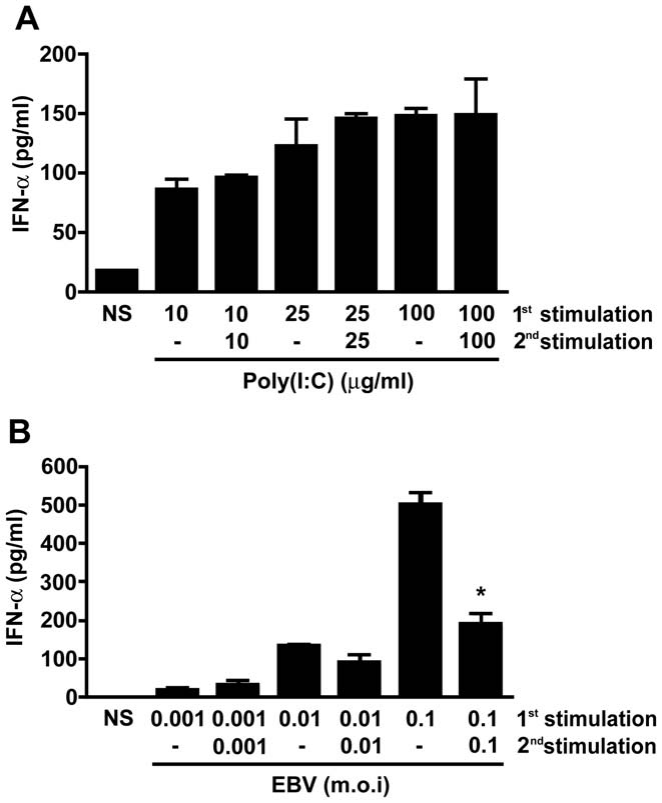
Effect of EBV infection on IFNα secretion by human monocytes. Monocytes (2×10^6^ cells) were stimulated with (**A**) poly(I:C) or (**B**) EBV for 20 hours. Following stimulation, cell-free supernatants were harvested (1^st^ stimulation). Cells were restimulated a second time with poly(I:C) or EBV for another 20 hours (2^nd^ stimulation) and cell-free supernatants were harvested for IFNα determination by ELISA. Data are representative of three independent experiments. *p≤0.05 when compared to EBV 1^st^ stimulation.

Upon recognition of pathogen-associated molecular patterns (PAMPs), several pattern-recognition receptors (PRRs) activate the production and secretion of type I IFN. The synthetic double-stranded RNA analog poly(I:C) is an agonist of both TLR3 and MDA-5 and is a known activator of type I IFN [22]. To study the secretion of IFNα by human monocytes in the absence of potential pathogen-derived inhibitory factor, we stimulated these cells either once or twice with various concentrations of poly(I:C). As shown in Figure 1A, a single stimulation with increasing concentrations of the agonist led to the secretion of IFNα in a dose-dependent fashion. When cells were stimulated a second time with the same concentrations of poly(I:C), IFNα levels did not significantly differ from what was observed after a single stimulation (Figure 1A). We repeated the experiment using live EBV and as observed with poly(I:C), a single monocyte treatment with increasing multiplicity of infection (m.o.i.) also led to increased IFNα secretion (Figure 1B). However, cells stimulated a second time with EBV secreted significantly less cytokine at an m.o.i. of 0.1 (Figure 1B). Given that monocytes did not become refractory to two stimulations with high concentrations of poly(I:C), these results are consistent with active interference on the IFNα secretion pathway caused by EBV infection.

### EBV infection induces the expression of SOCS proteins

**Figure 2:**
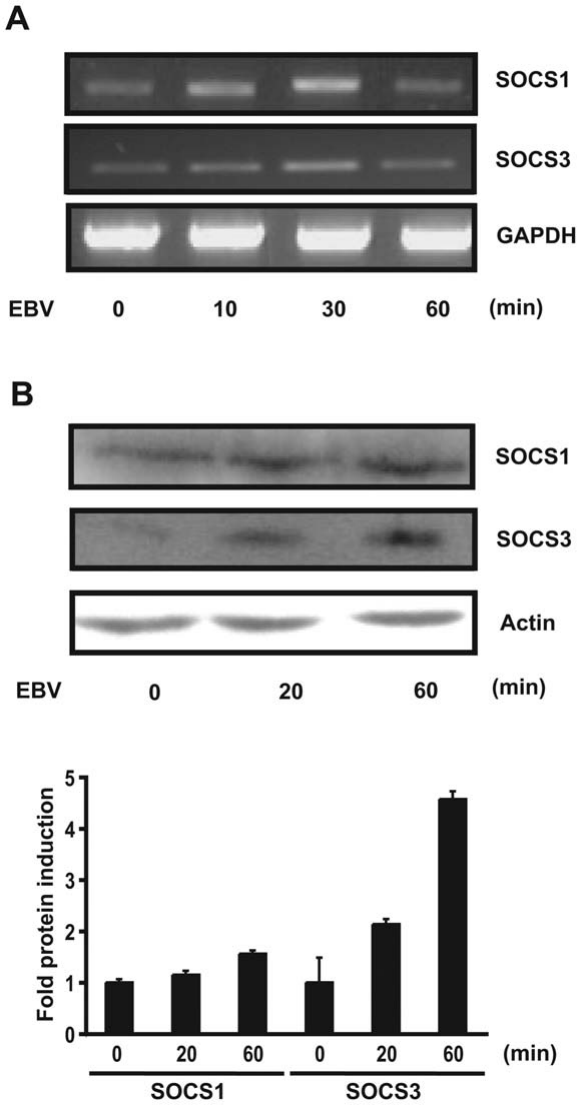
Expression of SOCS proteins following EBV infection of human monocytes. Monocytes (5×10^6^) were stimulated or not with EBV for the indicated time. (**A**) The expression of SOCS1 and SOCS3 proteins was evaluated by RT-PCR using described primers (Table 1) or (**B**) by Western blot analysis using specific anti-SOCS1 antibodies, anti-SOCS3 antibodies and anti-Actin as loading control. Densitometry was performed and represents fold protein induction (relative to 0 min.) ± std. dev. of experiments performed in duplicate. Data are representative of three independent experiments.

SOCS1 and SOCS3 are known to be involved in the negative feedback inhibition of IFNα signal transduction [14], [15]. Since we measured a decrease in IFNα secretion following a second monocyte infection with EBV, we wanted to investigate whether SOCS protein induction upon primary EBV infection might contribute to this observation. Monocytes were infected with EBV for various times and expression of SOCS1 and SOCS3 was evaluated at both the mRNA and protein levels. Transcription of both SOCS1 and SOCS3 was increased following EBV infection, reaching maximum levels after 30 minutes (Figure 2A). Increased SOCS expression was also confirmed at the protein level since SOCS1 expression was increased at 60 minutes post-infection whilst SOCS3 expression progressively increased from 20 to 60 minutes post-infection (Figure 2B). Thus, EBV infection causes the upregulation of two SOCS proteins involved in the modulation of the IFN pathway.

**Table 1:** RT-PCR primers used in this study.

Primer names	Sequences	Amplicon sizes
SOCS-1 sense	5′CACGCACTTCCGCACATTCC3′	300 bp
SOCS-1 antisense	5′TCCAGCAGCTCGAAGAGGCA3′	
SOCS-3 sense	5′TCACCCACAGCAAGTTTCCCGC3′	589 bp
SOCS-3 antisense	5′GTTGACGGTCTTCCGACAGAGATGC3′	
GAPDH sense	5′CCACCCATGGCAAATTCCATGGCA3′	598 bp
GAPDH antisense	5′TCTAGACG GCAGGTCAGGTCCACC3′	

### EBV-mediated SOCS3 activation causes the inhibition of the JAK/STAT pathway

**Figure 3:**
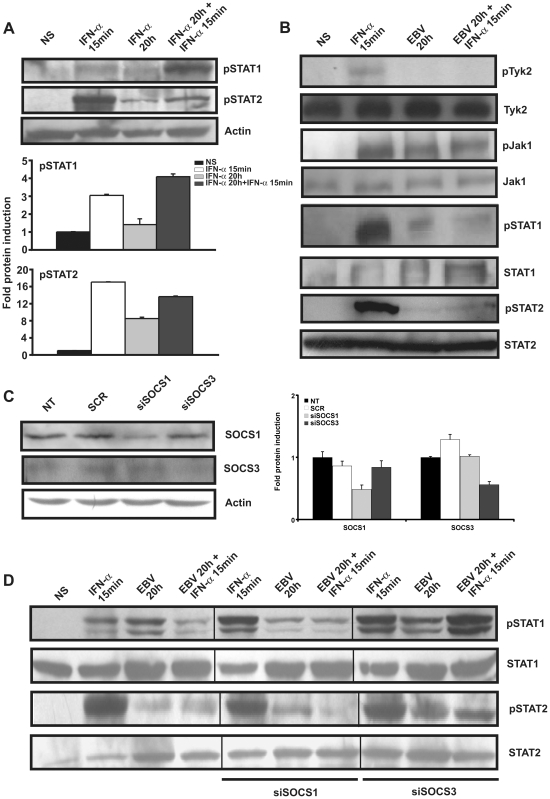
Effect of EBV infection on the activation of the JAK/STAT pathway. (**A**) Monocytes were incubated in the presence of IFNα (1000 U/ml) for 15 minutes and 20 hours. Following incubation (20 hours), cells were restimulated or not with IFNα for 15 minutes. The expression of phospho(p)STAT1, and phospho(p)STAT2 proteins was evaluated by Western blot analysis. Membranes were also probed with anti-Actin as a loading control. Densitometry was performed and represents fold protein induction (relative to non stimulated cells) ± std. dev. of experiments performed in duplicate. (**B**) Monocytes (5×10^6^) were treated with IFNα (1000 U/ml) for 15 minutes, with EBV for 20 hours or were pre-incubated for 20 hours in the presence of EBV followed by a stimulation with IFNα for 15 minutes. The expression of phospho(p)Tyk2, phospho(p)JAK1, phospho(p)STAT1, and phospho(p)STAT2 proteins was evaluated by Western blot analysis. Membranes were also probed with anti-Tyk2, JAK1, STAT1 and STAT2 as a loading control. (**C**) Monocytes (2×10^6^ cells) were transfected with 165 nM siRNA targeting SOCS1 or SOCS3 prior to EBV stimulation for 1 hour. Scramble siRNA was used as control. The expression of SOCS1 and SOCS3 was evaluated by Western blot analysis. Densitometry was performed and represents fold protein induction (relative to non-transfected cells) ± std. dev. of experiments performed in duplicate. (**D**) Monocytes (2×10^6^ cells) were either left untransfected or were transfected with siRNA targeting SOCS1 or SOCS3 and stimulated as in (B). The expression of phospho(p)STAT1 and phospho(p)STAT2 proteins was evaluated by Western blot analysis. Membranes were also probed with anti-STAT1 and STAT2 as a loading control. Data are representative of three independent experiments. NS: non-stimulated; NT: non-transfected; SCR: scrambled siRNA.

The cellular response to IFNα occurs via the JAK/STAT pathway downstream of the IFNα receptor [11]. To further dissect the response of monocytes to IFNα, we first monitored the phosphorylation of STAT1 and STAT2 in response to single or dual IFNα stimulation in the absence of viral infection. As shown in Figure 3A, a 15-minute stimulation with IFNα caused an increase in phospho-STAT1 and phospho-STAT2 levels. The amounts of phospho-STAT1 and phospho-STAT2 were both reduced following a prolonged 20-hour exposure to IFNα, as compared to a 15-minute stimulation only. Importantly, a 15-minute treatment with IFNα following a 20-hour exposure to IFNα caused an increase in both phospho-STAT1 and phospho-STAT2 compared to a 20-hour exposure only (Figure 3A). These results demonstrate that uninfected monocytes are still responsive to IFNα stimulation following prolonged exposure to this cytokine and establish a model system that can then be used to study the effect of EBV infection on the IFNα pathway.

SOCS1 and SOCS3 suppress IFNα signaling downstream of the IFNα receptor by blocking signal transduction through the JAK/STAT pathway [14]. To investigate whether EBV infection correlates with an impairment in JAK/STAT signaling downstream of the IFNα receptor, we first monitored the phosphorylation of Tyk2, Jak1, STAT1 and STAT2 in monocytes infected with EBV alone or infected and restimulated with IFNα. Triggering of the IFNα receptor through a short stimualtion with IFNα induced a strong activation of all members of the pathway (Figure 3B). Whilst infection of monocytes with EBV for 20 hours led to the phosphorylation of Jak1, STAT1 but not Tyk2 nor STAT2, increased phosphorylation of these proteins could not be observed upon restimulation of infected cells with IFNα (Figure 3B). Thus, EBV infection causes a blockage in the activation of the JAK/STAT pathway, a mechanism consistent with the action of SOCS1 and SOCS3.

To directly address the role of SOCS1 and SOCS3 in interfering with JAK/STAT signaling upon EBV infection, we used siRNA to silence the expression of these proteins. As shown in Figure 3C, both SOCS1- and SOCS3-targetting siRNA reduced expression levels of SOCS1 and SOCS3 respectively. Using both untransfected and transfected cells, we monitored phosphorylation of STAT1 and STAT2 under the same experimental conditions as in Figure 3B. In untransfected cells, akin to what was previously observed, a 15-minute IFNα stimulation of monocytes already infected with EBV for 20 hours did not increase levels of phospho-STAT1 and phospho-STAT2 as compared to EBV infected cells only (Figure 3D). Transfection of cells with siRNA against SOCS1 had no major effect on the phosphorylation of both proteins, however, inhibition of SOCS3 caused a marked increase in phospho-STAT1 and phospho-STAT2 in dually stimulated cells. Overall, these results indicate that EBV infection of monocytes causes the inhibition of the JAK/STAT pathway via SOCS3.

### Activation of IRF3 and IRF7 during EBV infection

**Figure 4:**
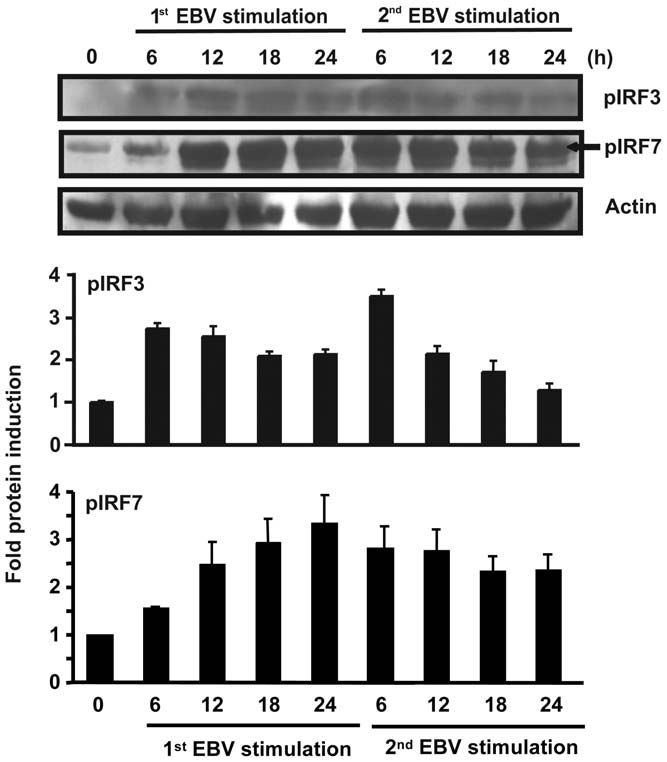
Effect of EBV infection on IRF3 and IRF7 activation. *1^st^ EBV stimulation*: Monocytes (5×10^6^ cells) were stimulated or not with EBV for the indicated times and expression of phosphorylated forms of IRF3 and IRF7 proteins was evaluated by Western blot analysis. *2^nd^ EBV stimulation*: Monocytes were first stimulated with EBV for 20 hours, washed and then restimulated a second time with EBV for the indicated times. Expression of phosphorylated forms of IRF3 and IRF7 proteins was evaluated by Western blot analysis. Membranes were also probed with anti-Actin as a loading control. Densitometry was performed and represents fold protein induction (relative to 0 h) ± std. dev. of experiments performed in duplicate. Data are representative of three independent experiments.

Although SOCS1 and SOCS3 are known to inhibit the JAK/STAT-mediated second wave of IFNα production, we were interested in whether IRF3 and IRF7, implicated in the first wave of type I IFN production, might also be affected by EBV infection. Indeed, it has been described that the JAK/STAT pathway modulates IRF7 expression via the formation of the interferon-stimulated gene factor 3 (ISGF3) complex [23], [24]. To verify the activation of both IRFs during primary EBV infection and upon restimulation, monocytes were stimulated once or twice with EBV and the presence of phosphorylated forms of IRF3 and IRF7 was evaluated by immunoblotting. Following a single stimulation with EBV, phospho-IRF3 and phospho-IRF7 were detected as early as 6 hours (Figure 4). Whilst levels of phospho-IRF3 decreased thereafter, phospho-IRF7 levels progressively increase from 6 to 24 hours. Upon a second stimulation with EBV, phospho-IRF3 was further induced after 6 hours and then progressively decreased to a much greater extent to what was observed during the first stimulation. However, in the case of phospho-IRF7, whilst levels of phosphorylation were detected after a second EBV stimulation, no further increase of phosphorylation levels was observed. These results suggest that IRF3 and IRF7 can be activated by EBV but that IRF7 activity can be progressively affected after a prolonged stimulation with EBV.

### SOCS3 plays a determinant role in the suppressive effect of EBV on IFNα secretion

**Figure 5:**
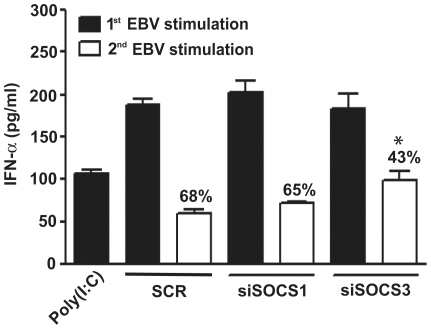
siRNA against SOCS3 restores IFNα secretion following EBV infection. Monocytes (2×10^6^ cells) were transfected with 165 nM siRNA targeting SOCS1 or SOCS3. Scramble siRNA was used as control. Twenty-four hours after transfection, cells were stimulated with EBV for 20 hours (*1^st^ EBV stimulation*). Following this first EBV stimulation, medium was replaced and cells were stimulated a second time with EBV (*2^nd^ EBV stimulation*) for an additional 20 hours. Cell-free supernatants were then harvested for IFNα determination. Data are representative of three independent experiments. Values in percentage represent the inhibition in IFNα secretion relative to the first respective EBV stimulation. *p≤0.05 compared to cells transfected with scramble siRNA and stimulated a second time with EBV. SCR: scrambled siRNA.

Our results highlighted a putative role for SOCS3 in the EBV-mediated suppression of IFNα secretion. To confirm its suppressive role, monocytes were transfected with siRNA directed against SOCS1 or SOCS3 and were infected once or twice with EBV. A first stimulation with EBV induced high levels of IFNα secretion, regardless of the siRNA transfected, as compared to poly(I:C) stimulation (Figure 5). When cells were stimulated a second time with EBV, the suppressive effect of the virus was detectable in control siRNA-transfected monocytes. Although SOCS1-targetting siRNA did not impact IFNα secretion after the second stimulation with EBV, inhibition of SOCS3 significantly restored cytokine secretion. These results confirm the direct involvement of SOCS3 in the suppressive effect of EBV on IFNα secretion in human monocytes.

### The viral protein Zta causes the transactivation of SOCS3

**Figure 6:**
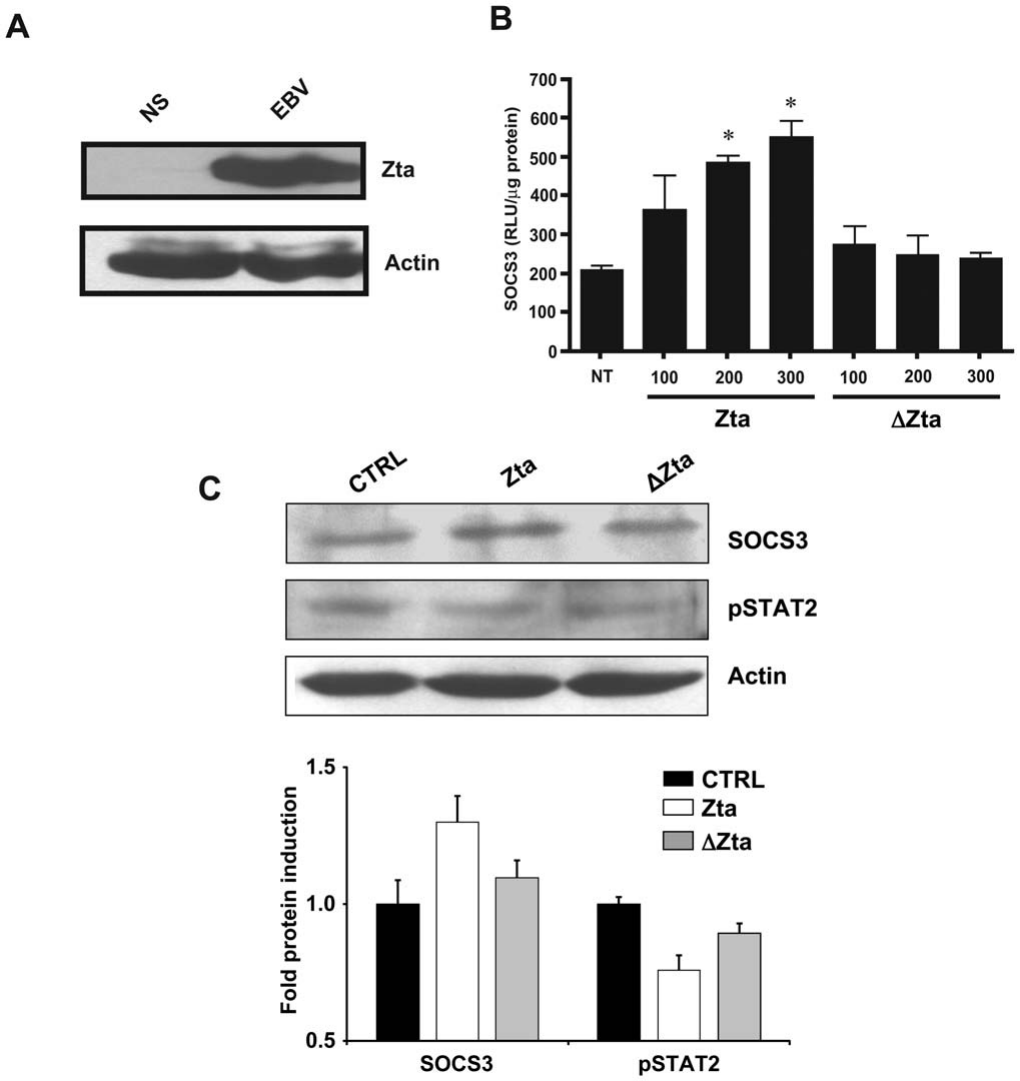
Activation of SOCS3 by the viral protein Zta. (**A**) Monocytes (5×10^6^) were mock- or EBV-stimulated for 20 hours and expression of the EBV protein Zta was evaluated by Western blot analysis. (**B**) HEK293 cells were transiently co-transfected with a vector encoding WT Zta or mutated Zta (ΔZta) at indicated concentrations along with a SOCS3 promoter-driven luciferase reporter vector. Luciferase assay was performed 48 hours post-transfection. (**C**) Monocytes (5×10^6^) were transfected with 300 ng of either Zta vector or ΔZta vector or mock vector control (CTRL). Forty-eight hours following transfection, monocytes were stimulated with IFNα (1000 U/ml) for 15 minutes. The expression of SOCS3 and phospho(p)STAT2 proteins was evaluated by Western blot analysis. Membranes were also probed with anti-Actin as a loading control. Densitometry was performed and represents fold protein induction (relative to mock-transfected cells) ± std. dev. of experiments performed in duplicate. Data are representative of two independent experiments. *p≤0.05 compared to non-transfected control cells. NS: non-stimulated; NT: non-transfected.

The EBV protein Zta is a basic leucine zipper (bZIP) transcription factor with many described functions including the interaction with host proteins and the modulation of cellular gene expression [25]. As shown in Figure 6A, Zta is strongly expressed in EBV-infected monocytes, further supporting the observation that EBV can efficiently infect this cell type [26]. In light of the many reports describing the modulation of immune-related host genes by Zta [25], [27], we wanted to investigate whether this viral transactivator could induce the expression of SOCS3. To do so, HEK293 cells were co-transfected with a reporter vector driven by the SOCS3 promoter along with either a vector encoding wild-type Zta (Zta) or a vector encoding a mutated form of Zta (ΔZta) that has lost its normal transactivation activity [28]. In this system, SOCS3 promoter activity was enhanced proportionally to the amount of transfected Zta vector, however, such activation was not observed using the ΔZta vector (Figure 6B). To confirm the ability of Zta to induce SOCS3 expression and modulate the JAK/STAT pathway, we transfected human monocytes with the Zta or the ΔZta vector or with a mock control prior to stimulation with IFNα and monitored levels of SOCS3 and phospho-STAT2 by immunoblot. The amount of SOCS3 protein was enhanced in cells transfected with the Zta vector as compared to the cells transfected with the ΔZta vector or the mock control (Figure 6C). In addition, increased SOCS3 expression in cells transfected with the Zta vector was accompanied by a marked decrease in phospho-STAT2 levels as compared to the cells transfected with the control vector (Figure 6C). Finally, we observed a partial restoration of phospho-STAT2 levels in cells transfected with the ΔZta vector. Thus, EBV protein Zta can transactivate SOCS3 expression in order to interfere with the IFNα response pathway in human monocytes.

## Discussion

**Figure 7:**
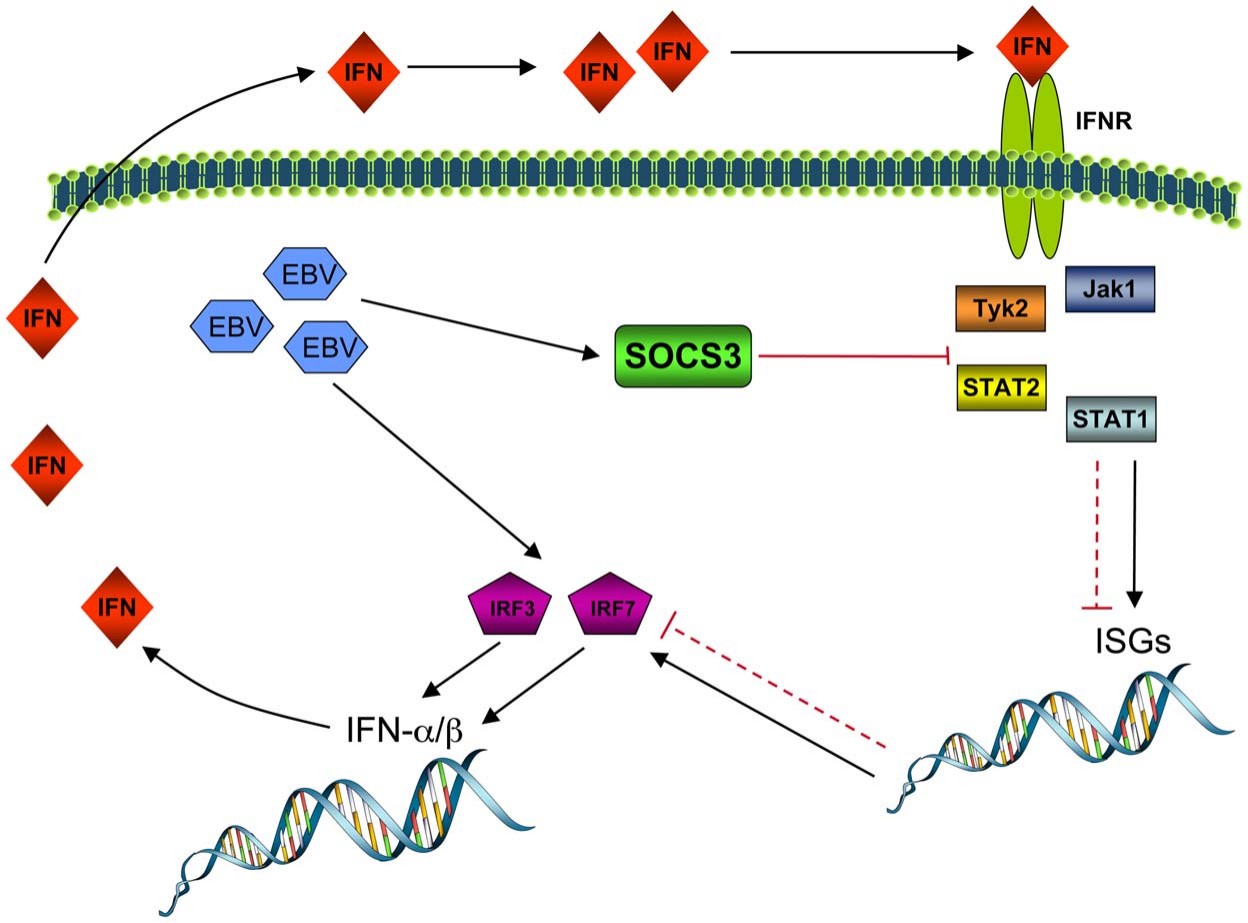
Proposed mechanism for EBV-mediated interference with type I IFN secretion by human monocytes. Following entry into the cell, EBV induces IRF3/7 activation leading to the initial production of IFNα/β. EBV infection also induces the synthesis of SOCS3 protein which results in inhibition of IFN receptor (IFNR) signaling and also in inhibition of the amplification of IFNα/β production. ISGs: interferon-stimulated genes. Black lines indicate “activation” and red lines indicate “suppression”. Red dotted lines represent suppressive effects of SOCS protein activation on JAK/STAT signaling events.

In the present study, we demonstrated that infection of primary human monocytes with EBV leads to the inhibition of the IFNα signal transduction pathway and hence, to an impairment in the amplification of IFNα secretion. Based on our results, we propose a hypothetical model of EBV-mediated negative regulation of IFN response and secretion in monocytes (Figure 7). According to this model, virion entry into the cell activates IRF3 and IRF7 leading to a first wave of type I IFN production. At the same time, EBV modulates SOCS3 expression in order to inhibit IFN receptor-mediated intracellular signaling through the JAK/STAT pathway. The latter results in a marked attenuation of the amplification loop initiated by the binding of type I IFNs to their cognate receptor. As a consequence, interferon-stimulated genes (ISGs) and IRF7 are negatively regulated and the second wave of IFNα secretion is impaired.

The importance of IFNs, originally discovered because of their ability to protect cells from viral infections, is highlighted by the observation that most viruses have evolved anti-IFN strategies [29]. Several studies have investigated mechanisms used by EBV to regulate the expression of IFNα and IFN-inducible genes. On one side, the early lytic EBV nuclear protein SM and the latent membrane protein 1 (LMP1) were reported to induce phosphorylation of STAT1 and the expression of ISGs [30], [31]. To counteract this cellular recognition event, EBV was shown to downregulate IFN-induced transcription via the viral protein EBNA-2 [32], [33] and to increase IFN receptor degradation via LMP2A and LMP2B [34] whilst EBV encoded EBER RNAs were found to be involved in IFN resistance by binding to PKR but failing to activate it [35], [36]. Another interesting mechanism of interference with IFN secretion was demonstrated by Cohen and Lekstrom who showed that EBV BARF1 gene (known to encode a soluble colony-stimulating factor receptor) inhibits IFNα secretion by mononuclear cells [37]. To our knowledge, we are the first to report SOCS protein activation during EBV infection of monocytes.

Viral-mediated induction of SOCS proteins is currently emerging as a key mechanism of immune evasion. Indeed, HSV-1, another member of the herpes virus family, has been shown to activate SOCS3 in infected epithelial cells leading to the downregulation of the JAK/STAT cascade [19]. The authors of the study concluded that HSV-1-induced SOCS3 was mainly responsible for the suppression of IFN signaling. In the case of EBV, we also observed that SOCS1 was induced in infected monocytes. Whilst the use of siRNAs directed against SOCS3 confirmed its role in EBV-mediated suppression of the JAK/STAT pathway and IFNα secretion, SOCS1 siRNA had no significant effect. The incomplete restoration of IFNα secretion with the use of SOCS3 siRNA shown in Figure 5 could either be explained by the difficulty to achieve high transfection efficiency in human monocytes coupled with the incomplete abolishment of SOCS expression by siRNA or by other viral-induced mechanisms targeting IFN signaling. Phosphatases such as protein tyrosine phosphatase 1B (PTP1B) [38] and SHP-2 [39] can interfere with the JAK/STAT pathway and represent candidate proteins potentially modulated by EBV. Other SOCS proteins such as CIS may also play a role. A recent study by Hashimoto *et al.*, investigated the induction of all eight SOCS proteins during RSV infection and found that SOCS1, SOCS3 and CIS were activated [21]. Suppression of the three proteins by siRNA inhibited viral replication and activated type I IFN signaling. Although we do not conclude that SOCS3 activation is sufficient for EBV-mediated interference with IFNα secretion, it does represent an important mechanism as demonstrated for HCV, HSV-1, enterovirus and RSV [17], [18], [19], [20], [21].

The transactivation of SOCS3 by Zta puts forward a new role for this viral effector protein. Zta is composed of a C-terminal transactivation domain, a central basic region that mediates DNA contact and a characteristic bZIP domain extending towards the N-terminus. Expression of Zta on its own is sufficient to disrupt EBV latency and this protein has a major role in EBV-associated cell transformation by modulating cellular gene expression and interacting with host cell-cycle proteins [25]. In our study, Zta was sufficient to induce SOCS3 expression and inhibit STAT2 phosphorylation upon IFNα stimulation of monocytes. Whilst SOCS3 expression could not be recapitulated with mutated Zta, STAT2 phosphorylation was only partly restored following IFNα treatment in this context. The ΔZta vector encodes the full-lenght Zta protein with two amino acid substitutions in the transactivation domain, only affecting part of its transcriptional activity [28]. Thus, ΔZta-mediated activation of other IFN signaling modulatory factors may account for the incomplete restoration of STAT2 phosphorylation. A possible factor is IL-10, which is known to be activated by Zta [40] and to inhibit IFNα-induced phosphorylation of STAT proteins [41]. Certainly, the pleiotropic action of Zta during EBV infection is only beginning to be fully revealed and its dual effects (activation/suppression) may be clarified through future investigations.

One example of such suppressive effect is the modulation of IRF7 by Zta. In a study by Hahn and colleagues, IRF7 activation was negatively regulated by Zta [42]. Zta did not affect IRF7 levels but expression of both IRF7 and Zta were found to be directly associated. Since Zta is a nuclear protein and that phosphorylated IRF7 translocates to the nucleus, the authors postulated that interaction between Zta and activated IRF7 might be responsible for downmodulating the transcription of IRF7 target genes. In our study, we monitored the phosphorylation status of endogeneous IRF3 and IRF7 in human monocytes. As opposed to phospho-IRF3, which could still be induced upon secondary stimulation with EBV, phospho-IRF7 progressively decreased under this condition. Based on our results, we suggest that the effect of Zta on IRF7 is indirect and implicates the inhibition of the JAK/STAT pathway by SOCS, thereby causing a decrease in ISGF3-driven IRF7 expression. It is interesting to note that whilst our proposed mechanism differs from that stated by Hahn *et al.*, both mechanisms are not mutually exclusive. As pointed out by the authors, IRF7, which was first cloned as a transcriptional regulator of the central EBV latency gene *EBNA-1*, is intricately associated with EBV infection [42]. Indeed, accumulating evidence highlights the use of different and/or redundant strategies by EBV to modulate IRFs expression and activity and interfere with the antiviral activity of type I IFNs [43], [44], [45]. Further research is needed to ask whether those strategies differ between cell types or upon primary EBV infection in comparison with reactivation from a latent infection.

Our study was performed using primary human monocytes in which productive EBV infection and viral-mediated alteration of several cellular functions have been demonstrated [3]. Here, we have shown that EBV infection induces SOCS3 activation via Zta and alters the IFNα signaling pathway. Using such a strategy, EBV might be able to survive longer within monocytes and optimize its dissemination. Furthermore, because monocytes are recognized as important antigen presenting cells linking the innate and adaptive immunity, suppression of their biological functions by EBV may thus affect the host immune response. Emerging therapeutic approaches aimed at downregulating SOCS gene expression [46] could possibly be beneficial against EBV infection by enhancing the innate antiviral activity of monocytes.

## Materials and Methods

### Ethics statement

Heparinized blood was obtained from healthy donors after written informed consent from all individuals in accordance with an Internal Review Board-approved protocol at CHUQ Research Center (Centre Hospitalier Université Laval).

### Isolation, purification and culture of human monocytes

Peripheral blood mononuclear cells (PBMCs) were isolated by centrifugation of heparinized blood obtained from healthy donors over Lymphocyte Separation Medium (Wisent Inc., St-Bruno, QC, Canada). PBMCs were next allowed to adhere onto autologous serum-treated petri dishes in order to separate monocytes from the lymphocyte population. Monocytes were further enriched by cell sorting (FACSAria, BD Biosciences, MD, USA) which resulted in at least 99% pure monocyte suspension as determined by flow cytometry analysis using anti-CD14 monoclonal antibodies. Cell viability was more than 99% as tested by trypan blue dye exclusion procedure. Isolated monocytes were resuspended in RPMI-1640 supplemented with 10% fetal bovine serum (FBS).

### Viral preparations

EBV strain B95-8 was produced as described previously [26]. Briefly, B95-8 cells were cultured in RPMI 1640 medium supplemented with 10% FBS in the presence of 20 ng/ml phorbol myristate acetate (PMA), a known inducer of viral reactivation. Cell-free supernatants were filtered through a 0.45 µm pore size filter and viral particles were concentrated by ultracentrifugation. Viral particles were resuspended in RPMI 1640 medium, titrated as described [47] and stored at −150°C until use. Cell-free supernatants collected from B95-8-infected cells not exposed to PMA were processed as described above and used as mock controls.

### Monocyte stimulation

Enriched monocytes were incubated with infectious EBV particles at the indicated multiplicity of infection (m.o.i.) or were transfected with poly(I:C) (Sigma-Aldrich, Oakville, ON, Canada) at indicated concentrations using lipofectamine reagent (Invitrogen, Burlington, ON, Canada) and cultured for 20 hours (first stimulation). Infected cells were then washed once in HBSS buffer and resuspended in fresh culture medium. Cells were then restimulated a second time with EBV, poly(I:C), or human IFNα (PBL Biomedical Laboratories, Piscataway, NJ) for indicated times (second stimulation). Following first and second stimulations, cell-free supernatants were harvested for IFNα quantitation by ELISA assay (PBL Biomedical Laboratories, Piscataway, NJ) or cells were lysed for Western blot or RT-PCR analyses as described below.

### RNA isolation and RT-PCR amplification

Untreated and EBV-treated monocytes were cultured for various periods of time before RNA extraction. Total RNA from monocytes was isolated using Trizol reagent (Invitrogen, Burlington, ON, Canada) according to the manufacturer's instructions. One microgram of DNase-treated RNA was reverse transcribed to cDNA with oligo (pdT) primers in a 20 µl reaction containing 20 U of SuperScript II RNase H Reverse Transcriptase and 1 U of RNase inhibitor (Invitrogen, Burlington, ON, Canada). A volume of 5 µl cDNA samples was subjected to 35 cycles of PCR amplification in 50 µl of PCR mixture containing 0.5 U of Taq DNA Polymerase and 1.5 µg of the appropriate primers. Primers used in this study are depicted in Table 1. GAPDH was used as internal control.

### Western blot analysis

Monocytes were incubated with appropriate agonists for indicated times, lysed (TAE buffer 1×, 1 mM EDTA, 27 mM sucrose, 1% Triton X-100) and boiled for 5 minutes after addition of sample buffer (150 mM Tris pH 6.8, 1.2% SDS, 0.33% glycerol, 15% β-mercaptonethanol, 1% bromophenol blue). Proteins were separated by SDS-polyacrylamide gel electrophoresis (SDS-PAGE), under reducing conditions followed by transfer onto a nitrocellulose membrane. Membranes were pretreated in blocking solution containing 5% (w/v) dry milk in Tris-buffered saline-Tween 20 for 1 hour at room temperature and then incubated overnight at 4°C with anti-pIRF3, anti-phospho or total JAK1, anti-phospho or total Tyk2, anti-phospho or total STAT1, anti-phospho or total STAT2, (Cell Signaling, Danvers, MA), anti-IRF3, anti-IRF7, anti-SOCS-1, anti-SOCS-3, or anti-β-actin (Santa Cruz Biotechnology, Santa Cruz, CA). Membranes were washed four times with Tris-buffered saline-Tween 20 and incubated either with HRP-conjugated sheep anti-mouse Ig or donkey anti-rabbit IgG antibodies (Jackson ImmunoResearch Laboratories Inc., West Grove, PA) for 1 hour. Immunoreactive proteins were revealed by enhanced chemiluminescence (Perkin Elmer, Woodbridge, ON, Canada). Densitometry analysis was performed using the Image J software and relative protein levels were normalized to relative β-actin levels.

### Small interfering RNA assay

Purified primary monocytes (2×10^6^ cells) were transfected with 165 nM of small interfering RNA against SOCS-1 (Sense: 5′-GCAUUAACUGGGAUGCCGUtt-3′ Antisense: 5′-ACGGCAUCCCAGUUAAU GCtg-3′) or SOCS-3 (Sense: 5′-GAACCUGCG CAUCCAGUGUtt-3′ Antisense: 5′-ACACUGGAUGCGCAGGUUCtt-3′) (Applied Biosystems/Ambion, Austin, TX) using lipofectamine according to the manufacturer's instruction. Scramble siRNA was used as control. Four hours post-transfection, cells were washed once in HBSS buffer and resuspended in culture medium in order to avoid cellular toxicity due to siRNA transfection. Twenty-four hours post-transfection, cells were stimulated as described and cell-free supernatants were harvested and tested for the presence of IFNα by ELISA or cells were lysed for Western blot analysis.

### Luciferase Assay and Plasmid Transfection

Human embryonic kidney (HEK293) cell line (ATCC, Manassas, VA) was cultured in Dulbecco modified Eagle medium (DMEM) supplemented with 10% heat-inactivated FBS. HEK293 cells (5×10^4^ cells/ml) were transiently co-transfected with 100, 200 or 300 ng of either pcDLSRα-Zta wt or pcDLSRα-Zta mutated (ΔZta) (Q34A and D35A) [28] plasmids kindly provided by Dr. Paul M. Lieberman using Escort transfection reagent (Sigma-Aldrich, Oakville, ON, Canada) along with 100 ng pGL3-pSOCS3 luciferase reporter plasmid. Forty-eight hours following transfection, cells were lysed in luciferase buffer (1% Triton, 10% glycerol, 20 mM Tris phosphate, pH 7.8) and luciferase activity was measured by luminometry. Relative light units (RLU) were normalized by protein dosage using BCA protein assay kit (Pierce Biotechnology, Rockford, IL). When indicated, monocytes (6×10^6^ cells/ml) were transfected with 300 ng of either pcDLSRα-Zta wt, pcDLSRα-Zta (ΔZta) or mock control (pcDL-SRα296) plasmids using Lipofectamine 2000 (Invitrogen, Burlington, ON, Canada). Four hours following transfection, monocytes were supplemented with 10% FBS. Forty-eight hours later, monocytes were stimulated with IFNα (1000 U/ml) for 15 minutes and expression of SOCS3 and phospho(p)STAT2 proteins was evaluated by Western blot analysis. Membranes were also probed with anti-Actin as loading control.

### Statistical analysis

Data were analyzed by one-tailed analysis of variance (ANOVA) followed by Newman-Kheuls post-hoc test using PRISM3 software. Differences were considered significant at p≤0.05.
